# Total flavonoids from Plumula Nelumbinis suppress angiotensin II-induced fractalkine production by inhibiting the ROS/NF-κB pathway in human umbilical vein endothelial cells

**DOI:** 10.3892/etm.2014.1554

**Published:** 2014-02-17

**Authors:** YONGWEN CAO, LULU ZHENG, SHAO LIU, ZHENYU PENG, SAIDAN ZHANG

**Affiliations:** 1 Department of Cardiology, Xiangya Hospital of Central South University, Changsha, Hunan 410008, P.R. China; 2 Department of Pharmacology, Xiangya Hospital of Central South University, Changsha, Hunan 410008, P.R. China; 3 Department of Emergency, The Second Xiangya Hospital of Central South University, Changsha, Hunan 410011, P.R. China

**Keywords:** total flavonoids from Plumula Nelumbinis, angiotensin II, reactive oxygen species, nuclear factor-κB, fractalkine, endothelial cells

## Abstract

Angiotensin II (Ang II) is a neuroendocrine factor that promotes hypertension and has been implicated in vascular inflammation through the induction of reactive oxygen species (ROS) and proinflammatory genes in endothelial cells. However, relatively little attention has been paid to the effect of Ang II on fractalkine (FKN), an important chemokine involved in endothelial dysfunction. In the study, we aimed to investigate the protective role of total flavonoids from Plumula Nelumbinis (TFPN), the main component extracted from Semen Nelumbinis, in Ang II-induced oxidative stress injury in human umbilical vein endothelial cells (HUVECs). Furthermore, we studied whether TFPN could attenuate the Ang II-induced generation of ROS and the activation of nuclear factor-κB (NF-κB); whether these Ang II-induced effects were inhibited by apocynin (a nicotinamide adenine dinucleotide phosphate oxidase inhibitor) and pyrrolidine dithiocarbamate (an NF-κB inhibitor). In the present study, it was observed that total flavonoids from Plumula Nelumbinis (TFPN), the main component extracted from Semen Nelumbinis, concentration-dependently inhibited the FKN production induced by Ang II in human umbilical vein endothelial cells (HUVECs). Furthermore, TFPN attenuated the Ang II-induced generation of ROS and the activation of nuclear factor-κB (NF-κB); these Ang II-induced effects were also inhibited by apocynin (a nicotinamide adenine dinucleotide phosphate oxidase inhibitor) and pyrrolidine dithiocarbamate (an NF-κB inhibitor). In conclusion, the findings of the present study indicate that TFPN attenuate Ang II-induced upregulation of FKN by inhibiting the ROS/NF-κB pathway in HUVECs and thus have a suppressive effect on vascular inflammation.

## Introduction

During the initiation and progression of hypertension, the vascular endothelium is constantly exposed to elevated angiotensin II (Ang II) levels, and certain endotheliocytes undergo oxidative stress, which contributes to endothelial dysfunction.

Reactive oxygen species (ROS), including the hydroxyl radical (HO^•^), superoxide anion (O_2_^−^) and hydrogen peroxide (H_2_O_2_), are continuously produced as a result of cellular oxidation-reduction processes stimulated by Ang II (1). Moreover, the high levels of Ang II-induced inflammatory factors may be mediated through oxidative stress (2). However, whether fractalkine (FKN), an important chemokine involved in endothelial dysfunction, is induced by Ang II remains unclear.

Plumula Nelumbinis is a type of traditional Chinese herbal medicine. The major components are alkaloids that exhibit a nerve-blocking effect. Thus, Plumula Nelumbinis may be used to treat hypertension (3). However, the role of the flavonoid components of Plumula Nelumbinis in the prevention of hypertension has not previously been studied. Flavonoids are present in a large number of plants and are strong antioxidants that scavenge various types of radicals through their H^+^-donating properties in aqueous and organic environments (4–8). Previous studies have indicated that flavonoids protect cells against ROS-induced inflammation by increasing the activity of antioxidant enzymes.

The present study investigated the protective role of total flavonoids from Plumula Nemlumbinis (TFPN) in Ang II-induced oxidative stress injury in human umbilical vein endothelial cells (HUVECs). To the best of our knowledge, this is the first time such a study has been conducted. In addition, the molecular mechanisms by which TFPN affect the production of ROS and malondialdehyde (MDA), the activity of superoxide dismutase (SOD) and the expression of nicotinamide adenine dinucleotide phosphate (NADPH) oxidase, IκB-α and FKN were investigated.

## Materials and methods

### Materials

Dulbecco’s modified Eagle’s medium (DMEM) and fetal bovine serum (FBS) were purchased from Hyclone (Logan, UT, USA). Penicillin and streptomycin were obtained from Gibco-BRL (Grand Island, NY, USA). The mouse anti-p47phox and anti-IκB-α primary antibodies were obtained from Anbo Biotechnology, Inc. (Sunnyvale, CA, USA). The mouse anti-FKN primary antibody and rabbit anti-mouse secondary antibodies were purchased from Santa Cruz Biotechnology, Inc. (Santa Cruz, CA, USA). The bicinchoninic acid (BCA) reagent, enhanced chemiluminescence (ECL) kit for western blotting and the fluorescent ROS detection kit were purchased from Beyotime Biotechnology (Shanghai, China). Reagent kits for measuring MDA and SOD were purchased from Jiancheng Bioengineering Institute (Nanjing, Jiangsu, China). Ang II, apocynin, pyrrolidine dithiocarbamate (PDTC), 3-(4,5-dimethylthiazol-2-yl)-2,5-diphenyl tetrazolium bromide (MTT) and dimethyl sulfoxide (DMSO) were purchased from Sigma-Aldrich (St. Louis, MO, USA). The TFPN were extracted from Plumula Nelumbinis by the Department of Pharmacology, Xiangya Hospital of Central South University (Changsha, China).

### Cell culture and treatment

HUVECs were purchased from the Cell Culture Center, Xiangya Medical College of Central South University. The HUVECs were cultured in DMEM supplemented with 10% FBS, 10 mmol/l HEPES, 100 U/ml penicillin and 100 mg/ml streptomycin at 37°C in a humidified atmosphere of 5% CO_2_ and 95% air. Endothelial cells in an actively growing condition from the fourth to sixth passages were used for the experiments. One day prior to treatment, 80–85% confluent cells were incubated with serum-free media for 24 h to synchronize cell growth. The cells were pretreated with the following drug interventions: 0.05, 0.1 or 0.2 mg/ml TFPN, 200 μM apocynin (an NADPH oxidase inhibitor) and 50 μM PDTC (an NF-κB inhibitor), for 4 h. Next, 10^−7^ mol/l Ang II was added to the medium containing the various drugs for an additional 24 h. Each treatment compound was individually dissolved in DMEM, and the cells cultured in the serum-free medium served as controls. The cells were incubated prior to protein isolation or the collection of cultured supernatant for chemical colorimetry. Each individual experiment was replicated at least three times.

### Effect of TFPN on HUVEC viability

The effect of TFPN on HUVEC viability was measured by the MTT assay, as previously described (9). HUVECs were counted and seeded into 96-well culture plates at a density of 0.6–1×10^4^ cells/well. The cells were pretreated with various concentrations of TFPN (0.001, 0.01, 0.025, 0.05, 0.1, 0.2, 0.4 or 0.6 mg/ml) for 4 h. Next, 10^−7^ mol/l Ang II was added to the medium, which was incubated for an additional 24 h. Each well was washed twice with PBS, then, for each well, 20 μl MTT solution (5 mg/ml) combined with 180 μl serum-free DMEM was added. Following incubation of the plate for 4 h, insoluble formazan crystals were dissolved in 150 μl DMSO. The optical density of each well was measured using an ELISA multiplate reader (Infinite 200 PRO multimode reader; Tecan Group Ltd., Männedorf, Switzerland) at 492 nm.

### MDA and SOD assays

The cells were cultured in 50-ml tissue culture flasks at a density of 5×10^5^ cells/ml and allowed to grow to confluence. Following cell treatment as described in Cell culture and treatment, supernatants of the cultures were collected for chemical colorimetry. The cells were washed with PBS, trypsinized for 1 min at 37°C, harvested by centrifugation (3,000 × g for 10 min) and resuspended in 2 ml PBS. Following ultrasonication at 4°C, the protein content was determined using the BCA method. The levels of MDA and SOD were determined using assay kits, according to the manufacturer’s instructions.

### Measurement of O_2_^−^ generation in intact cells

Changes in intracellular ROS levels were determined by measuring the oxidative conversion of 2′,7′-dichlorofluorescein diacetate (DCFH-DA; Invitrogen Life Technologies, Carlsbad, CA, USA), which is cell-permeable, to the fluorescent DCF in a SpectraMax M5 Muti-Mode Microplate Reader (Molecular Devices, Sunnyvale, CA, USA). The treated cells were washed with serum-free media and incubated with 10 mM DCFH-DA at 37°C for 20 min. DCF fluorescence was then detected using a fluorospectrophotometer. Fluorescence analysis was measured at 485 nm excitation and 535 nm emission. Fluorescence data are expressed as the percentage increase in fluorescence over that of untreated samples.

### Western blot analysis

The cells were lysed for 30 min at 4°C in a lysis buffer. The total cell protein concentration was determined using BCA reagent. Total protein (50 μg) was resolved by SDS-polyacrylamide gel electrophoresis, transferred to a PVDF membrane and subjected to immunoblot analysis. The primary antibodies for FKN (1:400), p47phox (1:500), IκB-α (1:500) and β-actin (1:1,000) and rabbit anti-mouse secondary antibodies were used. The bands were visualized using ECL reagents and the relative intensities were determined using a bio-imaging analyzer (ChemiDoc™ MP System 170-8280; Bio-Rad Laboratories, Inc., Hercules, CA, USA). The densities of the products were quantified using Image Lab 3.0^beta^ (version 3.0; Bio-Rad). All results were representative of at least three independent experiments.

### Statistical analysis

Data are expressed as the mean ± SD and were analyzed by ANOVA, followed by the Newman-Student-Keuls test for multiple comparisons. P<0.05 was considered to indicate a statistically significant difference.

## Results

### Effect of TFPN on HUVECs damaged by Ang II

**Figure 1:**
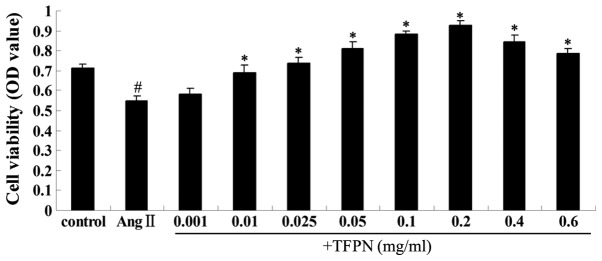
Effect of TFPN on the viability of HUVECs injured by Ang II. Cells were pretreated with various concentrations of TFPN (0.001, 0.01, 0.025, 0.05, 0.1, 0.2, 0.4 or 0.6 mg/ml) for 4 h. Ang II (10^−7^ mol/l) was subsequently added to the medium for an additional 24 h. Cell viability was measured by the MTT assay at 492 nm. ^#^P<0.01, vs. control; ^*^P<0.01, vs. Ang II; (n=4). +TFPN, Ang II + TFPN; TFPN, total flavonoids of Plumula Nelumbinis; HUVECs, human umbilical vein endothelial cells; Ang II, angiotensin II; MTT, 3-(4,5-dimethylthiazol-2-yl)-2,5-diphenyl tetrazolium bromide.

To evaluate whether TFPN protect against oxidative stress, HUVECs were pretreated with various concentrations of TFPN (0.001, 0.01, 0.025, 0.05, 0.1, 0.2, 0.4 or 0.6 mg/ml) for 4 h, and 10^−7^ mol/l Ang II was added to the medium for an additional 24 h. Next, cell viability was measured by the MTT assay. As shown in Fig. 1, Ang II markedly decreased the viability of the endothelial cells (P<0.01), while TFPN, at concentrations between 0.01 and 0.2 mg/ml, protected the cells from Ang II-induced cytotoxicity in a concentration-dependent manner (P<0.01). The lowest concentration of TFPN (0.001 mg/ml) did not have a protective effect against Ang II-induced damage on HUVECs (P>0.05). Higher concentrations of TFPN (0.4 and 0.6 mg/ml) increased the cell survival rate (P<0.01), but were slightly inferior to 0.2 mg/ml TFPN. The results indicate that TFPN protected HUVECs from Ang II-induced cellular injury.

### Effect of TFPN on the level of MDA

**Figure 2:**
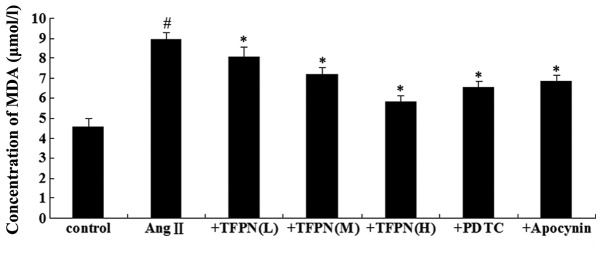
Effect of TFPN on the level of MDA. Cells were pretreated with various concentrations of TFPN (0.05, 0.1 or 0.2 mg/ml), 50 μM PDTC or 100 μM apocynin for 4 h. Ang II (10^−7^ mol/l) was added to the medium containing the various drugs for an additional 24 h prior to protein isolation. ^#^P<0.05, vs. control; ^*^P<0.05, vs. Ang II; (n=4). +TFPN(L), Ang II + 0.05 mg/ml TFPN; +TFPN(M), Ang II + 0.1 mg/ml TFPN; +TFPN(H), Ang II + 0.2 mg/ml TFPN; +PDTC, Ang II + PDTC; +Apocynin, Ang II + apocynin; TFPN, total flavonoids of Plumula Nelumbinis; MDA, malondialdehyde; Ang II, angiotensin II; PDTC, pyrrolidine dithiocarbamate.

To determine the effect of TFPN on the MDA level in Ang II-damaged endothelial cells, the cells were pretreated with TFPN, PDTC or apocyin for 4 h and then exposed to 10^−7^ mol/l Ang II for 24 h. As shown in Fig. 2, incubation of the cells with 10^−7^ mol/l Ang II increased the level of MDA (P<0.05). Compared with the MDA level in Ang II-treated cells, pretreatment with various concentrations of TFPN (0.05, 0.1 or 0.2 mg/ml) reduced the level of MDA in a concentration-dependent manner (P<0.05). Pretreatment with 50 μM PDTC and 100 μM apocynin also reduced the level of MDA (P<0.05).

### Effect of TFPN on SOD activity

**Figure 3:**
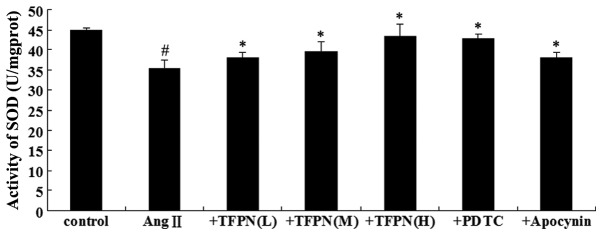
Effect of TFPN on SOD activity. Cells were pretreated with various concentrations of TFPN (0.05, 0.1 or 0.2 mg/ml), 50 μM PDTC or 100 μM apocynin for 4 h. Ang II (10^−7^ mol/l) was added to the medium containing the various drugs for an additional 24 h prior to protein isolation. ^#^P<0.05, vs. control; ^*^P<0.05, vs. Ang II; (n=4). +TFPN(L), Ang II + 0.05 mg/ml TFPN; +TFPN(M), Ang II + 0.1 mg/ml TFPN; +TFPN(H), Ang II + 0.2 mg/ml TFPN; + PDTC, Ang II + PDTC; +Apocynin, Ang II + apocynin; TFPN, total flavonoids of Plumula Nelumbinis; SOD, superoxide dismutase; Ang II, angiotensin II; PDTC, pyrrolidine dithiocarbamate.

To determine the effect of TFPN on SOD activity in Ang II-damaged endothelial cells, the cells were pretreated with TFPN, PDTC or apocyin for 4 h and then exposed to 10^−7^ mol/l Ang II for 24 h. As shown in Fig. 3, incubation of the cells with 10^−7^ mol/l Ang II decreased the SOD activity (P<0.05). Compared with the SOD activity in the Ang II-treated cells, pretreatment with various concentrations of TFPN (0.05, 0.1 or 0.2 mg/ml) increased the SOD activity in a concentration-dependent manner (P<0.05). Pretreatment with 50 μM PDTC and 100 μM apocynin also increased SOD activity (P<0.05).

### Effect of TFPN on ROS levels

**Figure 4:**
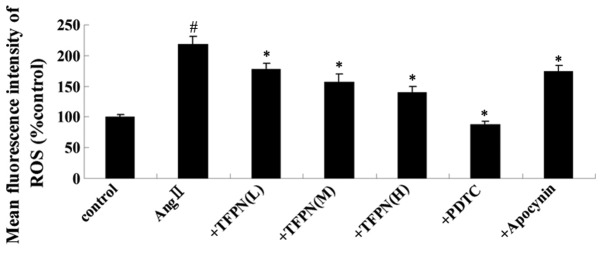
Effect of TFPN on the ROS levels. Cells were pretreated with various concentrations of TFPN (0.05, 0.1 or 0.2 mg/ml), 50 μM PDTC or 100 μM apocynin for 1 h. Ang II (10^−7^ mol/l) was added to the medium containing the various drugs for an additional 4 h prior to mean fluorescence intensity testing. ^#^P<0.01, vs. control; ^*^P<0.01, vs. Ang II; (n=4). +TFPN(L), Ang II + 0.05 mg/ml TFPN; +TFPN(M), Ang II + 0.1 mg/ml TFPN; +TFPN(H), Ang II + 0.2 mg/ml TFPN; +PDTC, Ang II + PDTC; +Apocynin, Ang II + apocynin; TFPN, total flavonoids of Plumula Nelumbinis; ROS, reactive oxygen species; Ang II, angiotensin II.

To determine the effect of TFPN on the level of ROS in Ang II-damaged endothelial cells, the cells were pretreated with TFPN, PDTC or apocyin for 4 h and then exposed to 10^−7^ mol/l Ang II for 24 h. As shown in Fig. 4, incubation of the cells with 10^−7^ mol/l Ang II increased the ROS levels (P<0.01). Pre-treatment with various concentrations of TFPN (0.05, 0.1 or 0.2 mg/ml) reduced the levels of ROS in a concentration-dependent manner compared with those in the Ang II-treated cells (P<0.01). Pretreatment with 50 μM PDTC and 100 μM apocynin also reduced the levels of ROS (P<0.01).

### FKN relative protein expression

**Figure 5:**
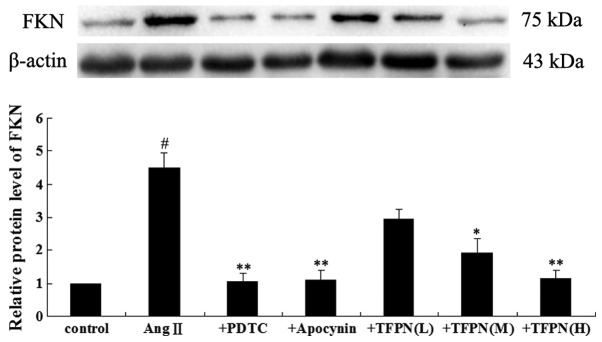
Cells were pretreated with 50 μM PDTC, 100 μM apocynin and various concentrations of TFPN (0.05, 0.1 or 0.2 mg/ml) for 4 h. Ang II (10^−7^ mol/l) was added to the medium containing the various drugs for an additional 24 h prior to protein isolation. The gels represent the western blot analysis results of FKN protein expression. β-actin was used as the internal control. ^#^P<0.01, vs. control; ^*^P<0.05, vs. Ang II; ^**^P<0.01, vs. Ang II; (n=3). +PDTC, Ang II + PDTC; +Apocynin, Ang II + apocynin; +TFPN(L), Ang II + 0.05 mg/ml TFPN; +TFPN(M), Ang II + 0.1 mg/ml TFPN; +TFPN(H), Ang II + 0.2 mg/ml TFPN; TFPN, total flavonoids of Plumula Nelumbinis; FKN, fractalkine; Ang II, angiotensin II; PDTC, pyrrolidine dithiocarbamate.

Western blotting was used to investigate whether FKN protein was upregulated in endothelial cells following treatment with Ang II, and to analyze whether TFPN inhibited this upregulation. As shown in Fig. 5, 10^−7^ mol/l Ang II significantly increased the protein expression level of FKN (P<0.01). With the exception of 0.05 mg/ml TFPN, pretreatment with TFPN (0.1 and 0.2 mg/ml) and pretreatment with 50 μM PDTC and 100 μM apocynin markedly blocked the Ang II-induced expression of FKN (P<0.05).

### p47phox relative protein expression

**Figure 6:**
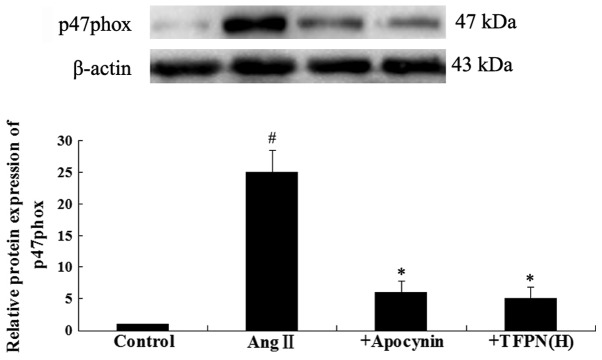
Cells were pretreated with 100 μM apocynin and 0.2 mg/ml TFPN for 4 h. Ang II (10^−7^ mol/l) was added to the medium for additional 24 h prior to protein isolation. The gels represent the western blot analysis results of p47phox protein expression. β-actin was used as the internal control. ^#^P<0.01, vs. control; ^*^P<0.05, vs. Ang II; (n=3). +Apocynin, Ang II + apocynin; +TFPN(H), Ang II + 0.2 mg/ml TFPN; TFPN, total flavonoids of Plumula Nelumbinis; Ang II, angiotensin II.

To clarify whether oxidase activation is involved in the effect of 0.2 mg/ml TFPN on Ang II-induced FKN elevation, western blotting for p47phox (a subunit of NADPH oxidase) was performed. As shown in Fig. 6, 10^−7^ mol/l Ang II significantly increased the protein expression of p47phox (P<0.01). Pretreatment with 0.2 mg/ml TFPN and 100 μM apocynin markedly blocked the Ang II-induced expression of p47phox (P<0.05).

### IκB-α relative protein expression

**Figure 7:**
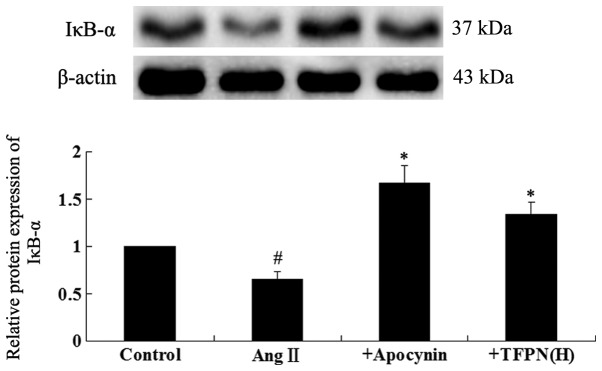
Cells were pretreated with 50 μM PDTC and 0.2 mg/ml TFPN for 4 h. Ang II (10^−7^ mol/l) was added to the medium for an additional 24 h prior to protein isolation. The gels represent the western blot analysis results of IκB-α protein expression. β-actin was used as the internal control. ^#^P<0.01, vs. control; ^*^P<0.01, vs. Ang II; (n=3). +PDTC, Ang II + PDTC; +TFPN(H), Ang II + 0.2 mg/ml TFPN; TFPN, Plumula Nelumbinis; Ang II, angiotensin II; PDTC, pyrrolidine dithiocarbamate.

To clarify the mechanism of the TFPN-associated reduction in FKN protein, western blotting for IκB-α (an endogenous inhibitor of NF-κB) was applied. As shown in Fig. 7, 10^−7^ mol/l Ang II significantly decreased the protein expression level of IκB-α (P<0.01). Pretreatment with 0.2 mg/ml TFPN inhibited the reduction of the IκB-α level induced by Ang II (P<0.01) and the same effect was also observed when the cells were pretreated with 50 μM PDTC (P<0.01).

## Discussion

The present study showed that Ang II activated p47phox and increased DCF-sensitive cellular ROS, MDA and FKN expression in HUVECs. However, SOD and IκB-α expression decreased following treatment with Ang II. The results of the present study indicate that the expression of FKN was regulated through the generation of NF-κB activated by ROS. The administration of each of TFPN, PDTC and apocynin alone significantly reduced the cellular oxidative responses to Ang II.

Endothelial dysfunction is the foundation and initial step of numerous cardiovascular diseases, and promotes abnormal vascular growth, leading to end-organ damage (10,11). However, the underlying molecular mechanisms remain poorly understood. Increasing evidence supports the critical roles of oxidative stress in endothelial dysfunction (12). Endothelial cells are involved in oxidative injury and serve as an important source of arterial oxidative stress (13). Endothelial oxidative stress increases cell apoptosis and the expression of inflammatory cytokine genes, inducing the arteries to fail, relax or dilate, resulting in increased tension of the arterial wall (14). Vascular smooth muscle cells proliferate due to endothelial dysfunction, which further increases the stiffness of the arteries (15). As a result, blood pressure increases.

Xanthine oxidase, uncoupled nitric oxide synthase and NADPH oxidase are likely potential endothelial ROS sources, with NADPH oxidase being the most significant (16). NADPH oxidase is an inducible electron transport system, which transfers reducing equivalents from NADPH to molecular oxygen via flavins, resulting in ROS generation (17). The expression and activation of NADPH oxidase has been demonstrated in a number of cell lines, including endothelial cells, vascular smooth muscle cells and cardiomyocytes (18). In endothelial cells, the upregulation of NADPH oxidase may be required for the expression of pro-inflammatory factors or chemokines induced by a neuroendocrine factor, such as Ang II (19).

The results of the present study showed that apocynin prevented the induction of FKN expression by Ang II in endothelial cells, indicating that FKN is responsive to NADPH oxidase activation with the potential involvement of ROS. PDTC was also found to reduce NF-κB-dependent FKN expression, which is consistent with results reported in previous studies (20–22). In endothelial cells, there are several transcriptional factor-binding sites in the cytokine promoter, including one for NF-κB (23). Activated NF-κB may bind to the cytokine promoter, which is critically involved in cytokine gene regulation by various stimuli, such as Ang II. ROS have been hypothesized to be secondary messengers leading to NF-κB activation in response to extracellular stimuli and then upregulating the gene expression of chemokines (24). Although PDTC is a well-known inhibitor of NF-κB, it has been shown that PDTC suppresses NF-κB activation partly through its antioxidant property, which may account for the reduced ROS formation (25). In the present study, Ang II increased the production of intracellular ROS and induced FKN expression in the endothelial cells; these effects were suppressed by antioxidant agents.

Flavonoids are broad-spectrum antioxidants and anti-inflammatory agents that are known to have efficacy in a number of inflammatory disease models, including coronary heart disease and hypertension (4–8). Moreover, the ethanol extracts from Plumula Nelumbinis have antioxidant and anti-inflammatory functions (26). The current study also indicated the antioxidant property of TFPN, which are the total flavonoids extracted from Plumula Nelumbinis. It was observed that TFPN inhibited the expression of the p47phox subunit of NADPH oxidase, ROS and MDA, while increasing the production of SOD, an oxyradical scavenger, and IκB-α, an endogenous NF-κB inhibitor. Oxygen radicals are hypothesized to participate in the regulation of NF-κB, inflammatory factors and chemokines through a NADPH oxidase-dependent pathway. Moreover, the results indicate that the effects of TFPN against NF-κB and FKN are associated with their antioxidant properties. The antioxidative effect of TFPN may be significant in the mechanism by which they prevent arterial inflammation and endothelial dysfunction.

The suppressive effects of TFPN on cellular oxidative stress and the inflammatory response to Ang II are significant. A series of studies regarded Ang II, which was selected as the cytokine stimulator of ROS in the present study, as one of the important inductive agents of oxidative stress and endothelial dysfunction *in vitro* and *in vivo* (1,2,27–29). ROS generation is one of the major mechanisms involved in Ang II-induced tissue damage (28). Ang II also contributes to ROS-dependent vascular smooth muscle cell proliferation in hypertension (29). These results indicate that the effects of Ang II on superoxide production are mediated through NADPH oxidase.

In the present study, TFPN were observed to attenuate the FKN expression induced by Ang II in endothelial cells in a concentration-dependent manner. Furthermore, TFPN decreased FKN expression at similar levels to apocynin and PDTC, indicating that the antioxidant effects of TFPN may be mediated via multiple mechanisms.

Inflammation and oxyradicals contribute to hypertension. FKN, a pleomorphic chemokine, contributes to endothelial dysfunction by inducing inflammatory responses in atherosclerotic disease (30). Previously, FKN levels were found to be increased in spontaneously hypertensive rats, indicating that FKN plays a role in hypertension (31). Furthermore, FKN has been shown to induce ROS production in arteries *in vitro* (32). The suppressive action of TFPN on FKN implies the prospect of the application of TFPN in the treatment of hypertension.

In conclusion, for the first time to the best of our knowledge, the present study reported that TFPN attenuate Ang II-induced ROS production and thus, ROS-induced NF-κB and FKN expression. TFPN exhibit these effects in HUVECs by a radical-scavenging mechanism through an NADPH oxidase-dependent process, indicating that TFPN may become a promising agent for the prevention of endothelial dysfunction.
